# Allele-Specific Up-Regulation of *FGFR2* Increases Susceptibility to Breast Cancer

**DOI:** 10.1371/journal.pbio.0060108

**Published:** 2008-05-06

**Authors:** Kerstin B Meyer, Ana-Teresa Maia, Martin O'Reilly, Andrew E Teschendorff, Suet-Feung Chin, Carlos Caldas, Bruce A. J Ponder

**Affiliations:** 1 Cancer Research UK, Cambridge Research Institute, Li Ka Shing Centre, Cambridge, United Kingdom; 2 Department of Oncology, University of Cambridge, Cambridge, United Kingdom; Johns Hopkins Medical School, United States of America

## Abstract

The recent whole-genome scan for breast cancer has revealed the *FGFR2* (fibroblast growth factor receptor 2) gene as a locus associated with a small, but highly significant, increase in the risk of developing breast cancer. Using fine-scale genetic mapping of the region, it has been possible to narrow the causative locus to a haplotype of eight strongly linked single nucleotide polymorphisms (SNPs) spanning a region of 7.5 kilobases (kb) in the second intron of the *FGFR2* gene. Here we describe a functional analysis to define the causative SNP, and we propose a model for a disease mechanism. Using gene expression microarray data, we observed a trend of increased *FGFR2* expression in the rare homozygotes. This trend was confirmed using real-time (RT) PCR, with the difference between the rare and the common homozygotes yielding a Wilcox *p*-value of 0.028. To elucidate which SNPs might be responsible for this difference, we examined protein–DNA interactions for the eight most strongly disease-associated SNPs in different breast cell lines. We identify two *cis*-regulatory SNPs that alter binding affinity for transcription factors Oct-1/Runx2 and C/EBPβ, and we demonstrate that both sites are occupied in vivo. In transient transfection experiments, the two SNPs can synergize giving rise to increased *FGFR2* expression. We propose a model in which the Oct-1/Runx2 and C/EBPβ binding sites in the disease-associated allele are able to lead to an increase in *FGFR2* gene expression, thereby increasing the propensity for tumour formation.

## Author Summary

Recently, a number of whole-genome association studies have identified genes that predispose individuals to common diseases such as cancer. The challenge now is to understand how the identified risk loci contribute to disease, since the majority of these loci are located within introns (which are discarded after transcription) and intergenic regions, and therefore do not change the coding region of nearby genes. This manuscript describes how two single–base pair changes in intron 2 of the *FGFR2* (fibroblast growth factor receptor 2) gene, “the top hit” of the breast cancer susceptibility study, exert their function. We find that the changes alter the binding of two transcription factors and cause an increase in *FGFR2* gene expression, thus providing a molecular explanation for the risk phenotype. This is the first functional study, to our knowledge, of the risk loci identified for breast cancer in a whole-genome scan and demonstrates that these studies can be used as valid starting points for studying the underlying biology of cancer.

## Abstract 3

Recent whole-genome scans have identified novel risk genes for many common diseases, challenging researchers to determine how these genes contribute to disease. A new study provides molecular insights into a breast cancer risk factor.

## Introduction

*FGFR2* (fibroblast growth factor receptor 2) plays a pivotal role both in mammary gland development and in cancer [1]. The *FGFR2* gene encodes a transmembrane tyrosine kinase and can function as a mitogenic, motogenic, or angiogenic factor, depending on the cell type and/or the microenvironment. Mammary epithelial cells express FGFR2IIIb (including alternatively spliced exon 9), which binds FGF-7 and FGF-10, which are normally expressed by surrounding mesenchymal cells. Mouse models of mammary carcinogenesis have long established the FGF signalling pathway as a major contributor to tumorigenesis [2], and a mouse mammary tumour virus (MMTV) insertional mutagenesis screen for genes involved in breast cancer has identified *FGFR2* and *FGF10* [3]. In human breast cancer, the expression of *FGFR2* has long been known to be elevated in estrogen receptor (ER)–positive tumours [4], which has been confirmed by data analysis performed with the ONCOMINE 3.0 array database [5,6]. Likewise both FGF-7 and FGF-10 have been found to be expressed in a proportion of breast cancers [7, 8]. Functional studies in cell lines have implicated FGFR2 as playing a role in tumourigenesis, with an alternative splicing in the C-terminal domain of FGFR2 giving rise to a more strongly transforming isoform [9]. However, as yet, nothing is known about the mechanism by which FGFR2 acts as a risk factor in predisposition to breast cancer.

We examined the functional implication of genetic variation in the *FGFR2* haplotype associated with susceptibility to breast cancer and we demonstrate increased gene expression for the risk allele.

## Results

**Figure 1:**
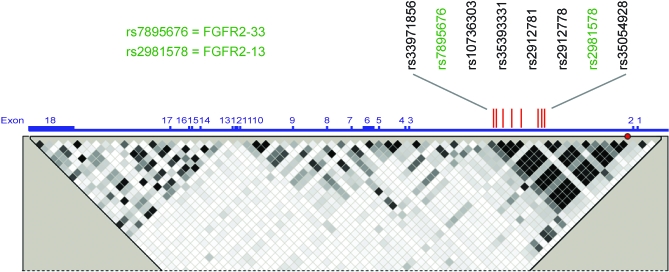
Diagram of the *FGFR2* Gene Genetic linkage is taken from HapMap, and the positions of the eight candidate SNPs (red lines) within intron 2 are indicated. The two SNPs for which data are presented in this study are shown in green. Red circle: original tagging SNP rs2981582.

**Figure 2:**
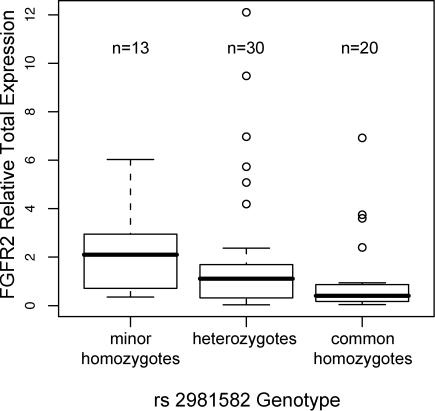
Correlation of *FGFR2* Expression in Breast Tumours with Genotype Quantitative RT-PCR was carried out using a probe targeting the 3' UTR of *FGFR2*.

Two independent studies have identified *FGFR2* as risk factor in breast cancer [10,11]. We have shown that in Europeans, the minor disease-predisposing allele of *FGFR2* is inherited as a haplotype of eight single nucleotide polymorphisms (SNPs) covering a region of 7.5 kb within intron 2 of the gene [10] (Figure 1), in a haplotype block with no linkage disequilibrium with the coding region of the gene. Microarray gene expression analysis on the Nottingham City Hospital cohort, using both the Agilent [12] and the Illumina [13] platforms, indicated that *FGFR2* is expressed at higher levels by tumours that are homozygous for the minor alleles than by those with the common alleles (Wilcox *p* < 0.05). Analysed tumours were all diploid for this region based on array-comparative genome hybridization data [14]. This correlation was independent of either ER expression or p53 mutation status of the cells. Quantitative TaqMan PCR analysis confirmed a significant increase in *FGFR2* expression in rare homozygotes, as compared to common homozygotes (Wilcox *p* = 0.028) (Figure 2). We also examined expression of the FGFR2 ligands FGF-7, FGF-10, and FGF-22, which are usually produced by the surrounding stroma, in 45 normal breast samples as well as the microarray data on tumours, but we found no correlation with genotype. Furthermore, *FGFR2* displays a very complex splicing pattern with the most commonly expressed variants of the N terminus of the gene either including exons 1, 2, and 3 or including exons 1 and 2, but lacking exon 3. Again, no correlation was observed between genotype and the presence or absence of exon 3. Thus, the risk genotype correlates with *FGFR2* expression itself, rather than affecting its function through receptor-ligand interactions.

**Figure 3:**
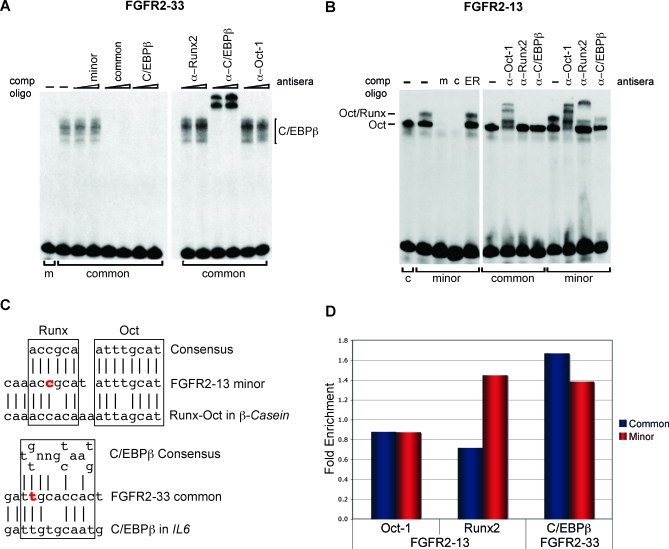
Protein–DNA Interactions at FGFR2–33 and FGFR2–13 In Vitro and In Vivo EMSAs on (A) FGFR2–33 and (B) FGFR-13 minor (m) and common (c) alleles, using 5 μg (FGFR2–33) and 2 μg (FGFR2–13) of HCC1954 nuclear extracts. Competitor oligonucleotides (minor, common, and ER as negative control) and antisera are indicated above each lane. (C) Alignment of the sequence around FGFR2–33 with binding site of C/EBPβ in the IL-6 promoter [15] and of FGFR2–13 with the Oct/Runx site in the β-casein gene [18]. The SNP is shown in red and the allele binding the transcription factor is shown. (D) ChIP assays for FGFR2–13 and FGFR2–33. Enrichment for the minor (HCC70^–/–^) and the common (T47D^+/+^) genotype is given relative to a negative control (TRXR2, located on 22q11.2) after normalisation against rabbit IgG.

This correlation suggests that the functional SNPs map to a regulatory region within the gene, possibly by altering one or more transcription factor binding sites. Interactions between proteins from nuclear extracts and DNA were examined for the eight most strongly disease-associated alleles (Figure 1). Two of these candidate functional SNPs showed distinct binding patterns in electrophoretic mobility shift assays (EMSA). The common allele of rs7895676 (FGFR2–33) formed strong protein–DNA complexes with nuclear extracts from the breast carcinoma cell lines HCC1954 (Figure 3A) and PMC42 and from HeLa cells (unpublished data), whereas no binding was detected on the minor allele. Competition studies and supershift experiments identify the bound protein as C/EBPβ (Figure 3A). We note that the FGFR2–33 sequence has considerable homology to the C/EBPβ binding site from the interleukin 6 (IL-6) promoter [15] (Figure 3C). The heterogeneity of the observed protein–DNA complexes is most likely due to the presence of multiple C/EBPβ isoforms. For rs2981578 (FGFR2–13), both alleles give rise to a strong protein–DNA complex in HCC1954 cell extracts. However, a second more slowly migrating complex was only seen on the rarer genotype (Figure 3B). Interestingly, both alleles are able to compete for both bands, suggesting that the formation of the upper complex depends on the presence of the lower complex. Inspection of the *FGFR2* DNA indicated the presence of a perfect octamer binding site immediately adjacent to the SNP, while the SNP itself lay within a sequence with homology to Runx binding sites (Figure 3C). Competition studies and incubation with specific antisera shows that both alleles bind Oct-1, while only the minor allele binds Oct-1 and Runx2 in HCC1954 nuclear extracts (Figure 3B), as well as in PMC42 cells (Figure S1).

To establish whether or not these sites were occupied in vivo, we carried out chromatin immunoprecipitation (ChIP) experiments using the ER^+^ breast cancer cell lines HCC70 and T47D, which are homozygous for the minor and the common *FGFR2* alleles, respectively. In addition, we confirmed that these cell lines were diploid for the *FGFR2* locus and only expressed the epithelial-specific isoform FGFR2IIIb [16]. The ChIP analysis was carried out on homozygous cell lines, because the SNP overlapping the C/EBPβ site lies in a repetitive region for which the different alleles could not be distinguished reliably by TaqMan PCR. A representative experiment is shown in Figure 3D. After Runx2-precipitation, the FGFR2–13 site is enriched 2-fold for the minor versus the common allele, confirming the EMSA results. Western blotting indicated that Runx2 is more abundant in T47D cells, thus confirming that differential ChIP in the two cell lines is due to the presence of the SNP. Oct-1 precipitation did not yield enrichment of FGFR2–13 for either allele. The Oct-1 epitope may either be sequestered within a higher-order complex or the antisera used do not work efficiently in a ChIP assay. On the FGFR2–33 site, we observed a 1.7-fold enrichment of C/EBPβ binding on the common allele. In addition, we observe that C/EBPβ can also bind to the minor allele, although less efficiently. Both cell lines contain comparable amounts of C/EBPβ as judged by Western blotting (unpublished data). In conclusion, both the C/EBPβ and the Runx2 binding sites are occupied in vivo.

**Figure 4:**
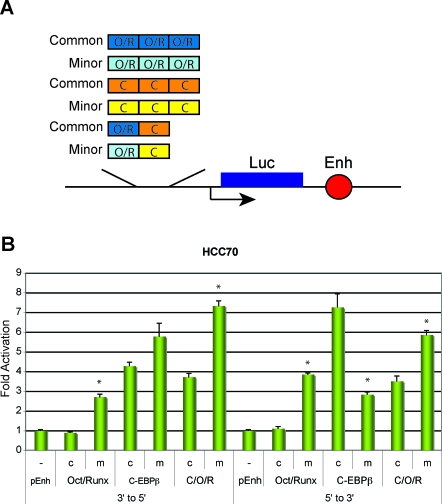
Transcriptional Activation by the Minor and Common Alleles of Oct-1/Runx2 and C/EBPβ Binding Sites of FGFR2 (A) Diagram of the concatemerised binding sites cloned into pGL3Enh. (B) Luciferase assays in HCC70 cells. Results are given as fold increase over pEnh activity. CMV-β-gal served as transfection control. The binding sites are indicated beneath each data point, with [Oct/Runx] and [C-EBPβ] being trimerized, while [C/O/R] contained only a single binding site for C/EBPβ, Oct-1, and Runx2. The asterisk denotes *p*-values <0.05 in a Student's *t*-test comparing the common versus the minor allele of each site.

To test whether differential protein binding could alter the ability of the susceptibility alleles to activate transcription, we multimerised oligonucleotides overlapping both the Oct-1/Runx2 and the C/EBPβ binding sites, cloned these in both orientations upstream of the luciferase reporter gene in pGL3Enh (Figure 4A), and assayed them in three breast cancer cell lines (PMC42, HCC70, and T47D). Transfections were carried out in triplicate and repeated at least twice for each cell line. A representative transfection into HCC70 cells is shown in Figure 4B (see Figure S2 for PMC42 and T47D). In all three cell lines tested, the minor allele at the Oct-1/Runx2 site stimulated transcription 2- to 5-fold over the common allele, independent of orientation, with the average being just above a 3-fold increase (*p* < 0.01). In contrast, the minor and common alleles of the multimerised C/EBPβ binding site did not show a consistent pattern of activation relative to each other. It varied with the cell lines and the orientation in which constructs were tested. Nevertheless, relative to the parental vector, the common allele always showed transcriptional activation. Compared to the common allele, the minor allele was either not significantly different or gave rise to a smaller degree of activation. However, in the latter case, the rare allele still activated transcription significantly above the enhancer-only construct (*p* < 0.01). Presumably this reflects the fact that the minor allele of FGFR2–33 still binds C/EBPβ above background levels in vivo (Figure 3D). By comparing the two different sites, we found that for Oct-1/Runx2 the minor allele was more active, while for C/EBPβ, the common site yielded higher levels of transcription in the majority of experiments. Hence their effects were opposing. We therefore assayed a synthetic construct consisting of single sites for C/EBPβ, Oct-1, and Runx2. In this arrangement, the effect of Oct-1/Runx2 clearly predominates, with the minor allele expressed at higher levels, reflecting the situation at the endogenous locus.

## Discussion

The data presented here lead us to conclude that the Oct-1/Runx2 binding site is the dominant determinant of differential expression between the common and minor haplotypes of *FGFR2*. Although Runx2 is a master regulator of osteoclast-specific transcription, Runx2 also plays an important role in mouse mammary gland–specific gene expression [17], where Runx2 activity is dependent on Oct-1 [18]. It is intriguing to note that in bone cells, overexpression of constitutively active *FGFR2* leads to increased levels of Runx2 mRNA [19]. *FGFR2* in turn is responsive to Runx2 in osteoclasts via the OSE2 (osteoclast specific element 2) in its promoter [20]. The description here of a Runx2 site in the *FGFR2* gene that is occupied in breast cancer cells, suggests that in the presence of the minor genotype, a similar positive feedback loop could also be established in breast cells. The role of the C/EBPβ binding site on *FGFR2* expression has been harder to define. The common allele binds C/EBPβ more tightly and activates transcription more strongly in most cases. Yet in a composite construct the activity of the Oct-1/Runx2 site dominates. This may be because C/EBPβ can directly bind to and synergize with Runx2 [21]. Thus, on the minor genotype, Oct-1 and Runx2 are present and able to synergize with the C/EBPβ bound (as suggested from the ChIP experiments), giving rise to higher levels of transcriptional activation. This is supported by the finding that a single copy of the C/EBPβ/Oct-1/Runx2 site gives rise to higher levels of activation than a concatemerized Oct-1/Runx2 site with six potential interaction sites (Figure 4A). A potential role for C/EBPβ in tumour etiology is supported by the observation that C/EBPβ is highly overexpressed in malignant human breast cells [22]. In conclusion, our evidence supports Oct-1/Runx2 as the probable primary determinant of activity, with C/EBPβ contributing to the risk haplotype.

The increased risk in breast cancer conferred by the *FGFR2* allele is predominant for ER^+^ breast tumours, while there is no significant increase in risk for ER^–^ tumours. Genome-wide analysis of ER binding sites has revealed three potential ER binding sites within the *FGFR2* gene [23], and ER and Oct-1/Runx2 may cooperate to increase gene expression. This is consistent with findings that Oct and ER sites often cluster [23]. The risk conferred by the disease-associated genotype may also depend on the signalling potential of *FGFR2* in ER^+^ cells. FGF-7 is over-expressed only in breast tumours that are ER^+^ [8]. Elevated levels of FGFR2 may then contribute to the establishment of an autocrine signalling loop, reducing the cell's propensity to undergo apoptosis [24]. Alternatively, paracrine signalling by mesenchymally or luminally derived FGF-7 or -10 on cells overexpressing FGFR2 may also drive cell proliferation.

To our knowledge, this is the first functional study on the risk loci recently identified for breast cancer. Our study demonstrates that SNPs identified by whole-genome scans can be used a valid starting points for studying the underlying biology of cancer. SNPs identified in other whole-genome scans for the genetic basis of complex diseases also primarily map in intronic or intergenic regions. Our observation that an identified SNP regulates the expression of the risk allele is therefore likely to be a common theme. Breast cancer is one of the most common cancers in the developed world. The *FGFR2* minor allele carries only a small increase in risk and acts as part of a spectrum of risk factors. However, it has a high minor allele frequency (0.4), and *FGFR2* is therefore likely to contribute to the incidence of breast cancer in many individuals.

## Materials and Methods

### Genotyping.

DNA from the 170 tumour samples was genotyped using a fluorescent 5′ exonuclease assay (TaqMan) and the ABI PRISM 7900 Sequence Detection Sequence (PE Biosystems) in 384-well format. Duplicate samples were included to assess concordance and quality of genotyping. The genotyping assay was designed for rs2981582, which tags the whole haplotype block associated with the disease [10].

### Analysis of *FGFR2* gene expression.

Analysis was performed on total RNA from breast tumour cases. cDNA was prepared with the TaqMan Reverse Transcription Reagents kit (Applied Biosystems) using random hexamers, according to the manufacturer's instructions. Expression levels were determined using a TaqMan Gene Expression Assay (Hs00240796_m1, Applied Biosystems) and normalized to four different housekeeping genes.

### Statistical analysis.

To assess whether there were significant statistical differences between the expression levels across the genotype groups we used a Wilcoxon test, fitted using the R statistical framework. Elsewhere, Student's *t*-tests were carried out using Microsoft Excel.

### Cell lines and cell culture.

Breast cancer cell lines HCC1954, HCC70, T47D, and PMC42 were cultured in RPMI supplemented with 10% foetal calf serum and penicillin/streptomycin under standard conditions. These cell lines have been characterised extensively, and karyotypes are available at the Cancer Genomics Program of the University of Cambridge (http://www.path.cam.ac.uk/~pawefish).

### EMSAs.

Small-scale nuclear extracts and bandshifts were carried out as previously described [25], except that Complete Protease Inhibitors (Roche) were used. In supershift experiments, polyclonal antisera against Oct-1 (sc-232x), Runx2 (sc-10758x), and C/EBPβ (sc-150x) were obtained from Santa Cruz Biotechnology, Inc and up to 8 μl were added per reaction, unless otherwise stated. Oligonucleotides (Table S1) were annealed to complementary strands, and the resulting BamHI overhangs filled in with Klenow enzyme, using radiolabelled [α^32^P]dCTP (GE Healthcare, UK).

### ChIP.

Primers were designed using Primer Express (Applied Biosystems) and Lasergene (DNA Star) to amplify regions of up to 100 bp comprising the SNPs of interest, plus one negative control (region of the genome not suspected to bind any of the transcription factors of interest) (Table S1). PCR amplification was carried out with Power SYBR Green Mastermix (Applied Biosystems), using 2 μl of precipitated and purified DNA as described [23]. The antisera were as in the EMSAs, except for C/EBPβ, which was a polyclonal serum from Abcam, UK.

### Plasmid construction and luciferase assays.

The pGL3-Enhancer vector (Promega) was linearized with BglII and re-circularised in the presence of annealed oligonucleotides (Table S1). All constructs were verified by sequencing. DNA was prepared using Qiagen kits and transfected into tumour cell lines cultured in 24-well plates. Per well, 500 ng of reporter and 100 ng CMV-β-galactosidase plasmid were tranfected using 2 μl of Fugene 6 (Roche), harvested 36–48 h later and extracts prepared using 100 μl Promega lysis buffer. Luciferase and β-galactosidase activity in 25 μl was measured using Promega reagents. Results are given as ratios of luciferase over β-galactosidase activity.

## Supporting Information

Figure S1 EMSA on the Common and Minor Allele of FGFR2–13 using PMC42 Nuclear Extracts 5 μg of nuclear extract and 8 μl of α-Oct-1 (ab15112), α-Runx2 (ab11906), and α-C/EBPβ (ab32358) from Abcam, UK, were included as shown above the lanes. ns, non-specific binding.
(3.04 MB AI).

Figure S2 Transcriptional Activation by the Minor and Common Alleles of Oct-1/Runx2 and C/EBPβ binding sites of FGFR2 (A) Diagram of the concatemerised binding sites cloned into pEnh. These constructs were assayed in the cell lines.
(B) PMC42 and (C) T47D cells. Results are given as fold increase over pEnh activity. CMV-β-galactosidase served as transfection control. The binding sites are indicated beneath each data point, with [Oct/Runx] and [C-EBP] being trimerized, while [C/O/R] contained only a single binding site for C/EBPβ, Oct-1, and Runx2.
(238 KB PPT)

Table S1 Oligonucleotides Used in This Study. Sequences in brackets show the two alleles (common/minor) of SNPs.
(50 KB DOC)

## Abbreviations

ChIP: chromatin immunoprecipitation
EMSA: electrophoretic mobility shift assay
ER: estrogen receptor
FGFR2: fibroblast growth factor receptor 2
SNP: single nucleotide polymorphism
