# Supplementary material for: Allele-Specific Up-Regulation of FGFR2 Increases Susceptibility to Breast Cancer
Source: PLoS Biol. 2008 May 6;6(5):e108. doi: 10.1371/journal.pbio.0060108 (PMC2365982; doi:10.1371/journal.pbio.0060108)
Supplement: Supplementary file 1 [file pbio.0060108.sg001.pdf]

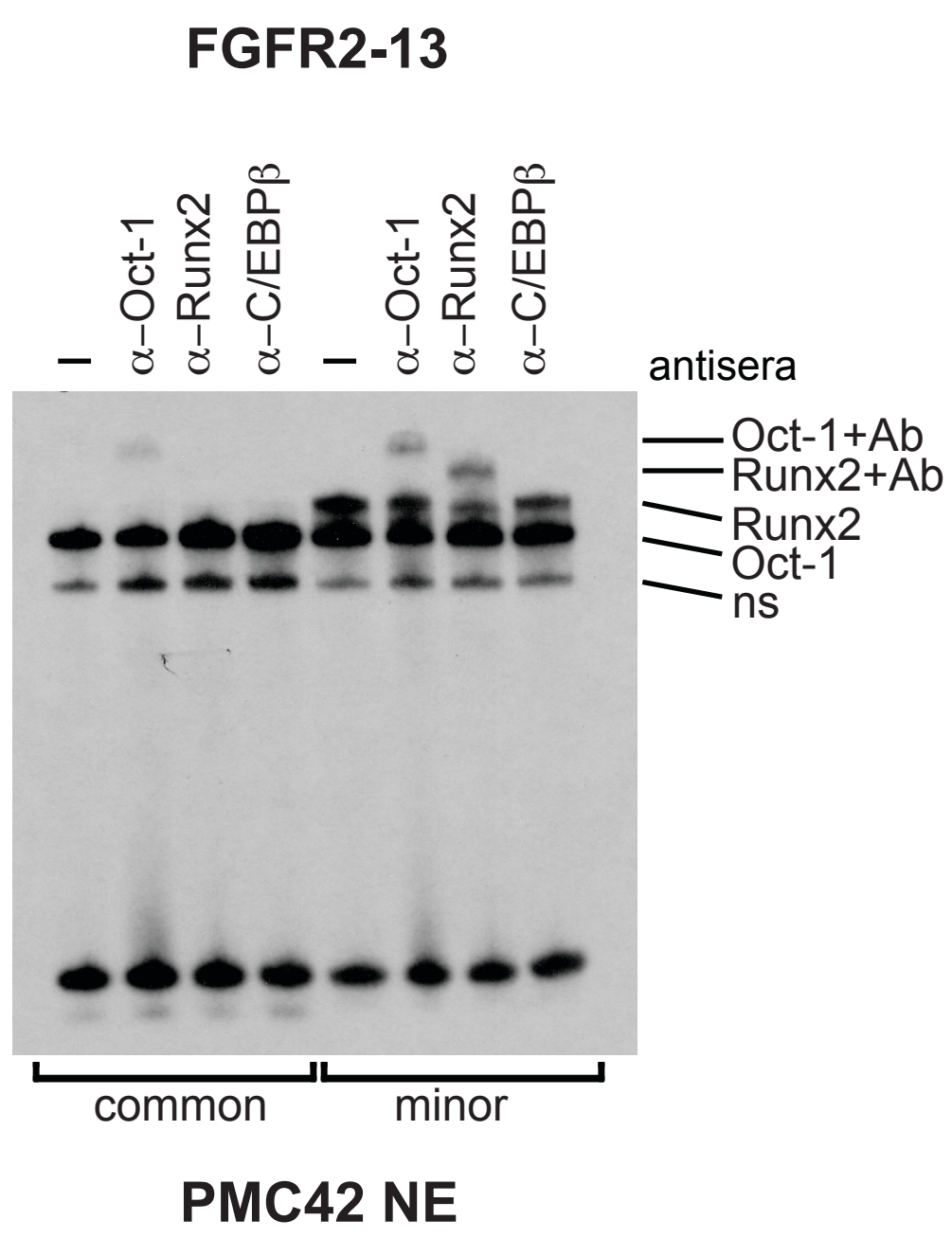
FGFR2-13
–
α–Oct-1
α–Runx2
α–C/EBPβ
–
α–Oct-1
α–Runx2
α–C/EBPβ
antisera
Oct-1+Ab
Runx2+Ab
Runx2
Oct-1
ns
common
minor
PMC42 NE
