# Supplementary material for: Allele-Specific Up-Regulation of FGFR2 Increases Susceptibility to Breast Cancer
Source: PLoS Biol. 2008 May 6;6(5):e108. doi: 10.1371/journal.pbio.0060108 (PMC2365982; doi:10.1371/journal.pbio.0060108)
Supplement: Supplementary file 2 [file pbio.0060108.st001.doc]

| **EMSA** |  |
| --- | --- |
| FGFR2-33  FGFR2-13  C/EBPβ (IL-6)  Vκ Oct  Runx  SRF  Oct/Runx  C/EBPβ | gcagtgagcccagat(t/c)gcaccactgcactc  ctctatgcaaatatgc(a/g)gtttggagcaggg  gttattaagattgtgcaatgtgacgt  gccttcgggtatgcaaattattaagtct  gaactgaccgcagctggccgt  gccccttacacaggatgtccatattaggact  gatct[tccaaac(a/g)gtttgga]_3_a  gatct[gcccagat(t/c)gcaccactgca]_3_a |
| **Luciferase Assays** |  |
| Oct/Runx  C/EBPβ  EBP/O/R | gatct[tccaaac(a/g)gtttgga]_3_a  gatct[gcccagat(t/c)gcaccactgca]_3_a  gatccgcccaga(t/c)gcaccactgcacctcatgcaaatatgc(a/g)tttggaa |
| **Genotyping** |  |
| rs2981582 | Genotyping Assay-by-Design (ABI) |
| **ChIP** |  |
| FGFR2-33 FW  FGFR2-33 REV  FGFR2-13 FW  FGFR2-13 REV  IL-6 FW  IL-6 REV  Casein FW  Casein REV  TRXR2 | caaaaattagatgggcatggtggtgtg  ggttctgacttcaatggcgggactc  aggtagtgactcttcaaagtttgtttgt  cgccatcacagttaacctttcttc  cttttccccctagttgtgtcttg  ttgataaatctttgttggagggtgag  ttggacagaaaattagtttcc  gaagcaagttgtgttgatgac  Genotyping Assay-by-Design (ABI) |
